# Ginsenoside Rh2 Induces HeLa Apoptosis through Upregulating Endoplasmic Reticulum Stress-Related and Downstream Apoptotic Gene Expression

**DOI:** 10.3390/molecules27227865

**Published:** 2022-11-14

**Authors:** Ying Liu, Xinran Wang, Juhui Qiao, Jiawen Wang, Leilei Jiang, Chenxi Wang, Shiting Yu, Peiguang Zhang, Daqing Zhao, Meiling Fan, Meichen Liu

**Affiliations:** 1 Northeast Asia Research Institute of Traditional Chinese Medicine, Changchun University of Chinese Medicine, Changchun 130117, China; 2 Changchun Institute of Optics, Fine Mechanics and Physics, Chinese Academy of Sciences, Changchun 130033, China; 3 Department of Obstetrics and Gynecology, Changchun University of Chinese Medicine, Changchun 130117, China

**Keywords:** ginsenoside Rh2, apoptosis, RNA-seq, cervical cancer, molecular docking, endoplasmic reticulum stress

## Abstract

Cervical cancer is a common gynecological malignancy afflicting women all over the world. Ginsenoside Rh2 (GRh2), especially 20(S)-GRh2, is a biologically active component in the natural plant ginseng, which can exhibit anticancer effects. Here, we aimed to investigate the effect of 20(S)-GRh2 on cervical cancer and elucidate the underlying mechanism through RNA-seq. In this study, the CCK-8 assay showed that 20(S)-GRh2 inhibited HeLa cell viability in a time- and dose-dependent manner. Caspase 3 activity and Annexin V staining results showed that 20(S)-GRh2 induced apoptosis of HeLa cells. Gene function enrichment analysis revealed that the biological process gene ontology (GO) terms were associated with the apoptotic signaling pathway. Biological process GO terms’ similarity network indicated that apoptosis might be from endoplasmic reticulum stress (ERs). Kyoto Encyclopedia of Genes and Genomes enrichment analysis revealed that 20(S)-GRh2 primarily modulates apoptosis pathway genes. Combined protein–protein interaction network, hub gene screening, and qPCR validation data showed that ERs-related genes (ATF4 and DDIT3) and the downstream apoptotic genes (JUN, FOS, BBC3, and PMAIP1) were potential novel targets of 20(S)-GRh2-inducing cervical cancer cell apoptosis. Differential transcript usage analysis indicated that DDIT3 is also a differential transcript and its usage of the isoform (ENST00000552740.5) was reduced by 20(S)-GRh2. Molecular docking suggested that 20(S)-GRh2 binds to the targets (ATF4, DDIT3, JUN, FOS, BBC3, and PMAIP1) with high affinity. In conclusion, our findings indicated that 20(S)-GRh2 might promote ERs-related apoptosis of cervical cancer cells by regulating the DDIT3-based targets’ signal pathway. The role of 20(S)-GRh2 at the transcriptome level provides novel targets and evidence for the treatment of cervical cancer.

## 1. Introduction

Cervical cancer (CC) is a common gynecological malignant tumor that ranks fourth in morbidity and mortality among all cancers in women worldwide [1]. Surgery is currently the best treatment option for patients at an early stage of CC [2]. However, despite treatment with surgical resection with adjuvant radiotherapy and chemotherapy, the prognosis of patients with advanced CC is still poor [3]. The side effects of traditional chemotherapeutic drugs and the acquired drug resistance of tumors further support the need to identify new efficient antitumor drugs with low toxicity [4].

Natural products have attracted much attention as cancer treatments because of their strong potency and low toxicity, and several products have shown efficacy in cancer control and treatment [5,6]. Ginsenoside Rh2 (GRh2) is the natural extract of Panax ginseng C.A. Meyer (ginseng) and exhibits biological activities in inhibiting cell proliferation and inducing cell apoptosis in oral cancer, colon cancer, and prostate cancer [7,8,9]. GRh2, a major deglycosylated metabolite of ginsenoside Rg3, Rb1, Rb2, and Rc, exhibits stronger tumor-suppressive effects than the parent saponin, along with good biosafety and low molecular weight [10,11]. It is noteworthy that the molecular structure of GRh2 has R-type and S-type, and the 20(S)-GRh2 configuration plays the main anticancer effect [12]. Thus, application of 20(S)-GRh2 may be a promising anticancer therapeutic strategy. However, the underlying targets of 20(S)-GRh2 anticervical cancer remained elusive.

Herein, we performed transcriptome profiling (RNA-Seq) to seek potential targets in 20(S)-GRh2-treated HeLa cells, with the aim of elucidating the underlying mechanisms of 20(S)-GRh2.

## 2. Results

### 2.1. 20(S)-GRh2 Induces HeLa Cell Apoptosis

**Figure 1:**
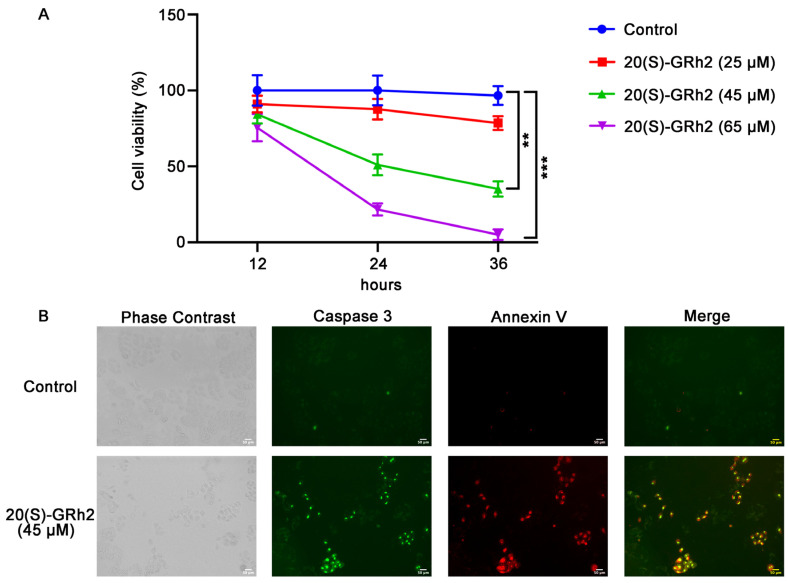
20(S)-GRh2 inhibited HeLa cell proliferation and induced HeLa cell apoptosis. (**A**) HeLa cells were treated with control (0.1% *v*/*v* DMSO) or 25, 45, 65 µM 20(S)-GRh2 at different time intervals (12 h, 24 h, 36 h), and CCK-8 assay was used to detect cell viability. Data are presented as mean ± standard deviation of three independent experiments (two-way ANOVA, ** *p* < 0.01, *** *p* < 0.001 vs. control). (**B**) Images of HeLa cells treated with 45 µM 20(S)-GRh2 or control (0.1% *v*/*v* DMSO) and examined using the Live Cell Caspase-3 Activity and Annexin V Apoptosis Detection reagent. Green fluorescence represents caspase 3 activity, red fluorescence represents apoptotic cells, and phase contrast shows the total cells. The images are representative from three independent experiments. PS, phosphatidylserine; 20(S)-GRh2, 20(S)-ginsenoside Rh2.

20(S)-GRh2 treatment resulted in marked inhibition (IC_50_ of approximately 45 μM at 24 h) of HeLa cell proliferation in a dose- and time-dependent manner (Figure 1A). To avoid an excessive response of cells to drug toxicity, we treated with 45 μM 20(S)-GRh2 continuously for 24 h in subsequent experiments.

To explore whether 20(S)-GRh2-mediated inhibition of proliferation in HeLa cells involved apoptosis, cells were treated with 20(S)-GRh2 and examined using caspase 3 substrate and Annexin V-mCherry. As shown in Figure 1B, a significant increase in apoptosis, determined by increased caspase 3 activity and phosphatidylserine (PS) eversion, was observed in HeLa cells treated with 20(S)-GRh2 compared with the control group. These results demonstrated that 20(S)-GRh2 inhibits cell proliferation of HeLa cells via inducing apoptosis.

### 2.2. Identification of Differentially Expressed Genes (DEGs) in 20(S)-GRh2-Treated HeLa Cells

**Figure 2:**
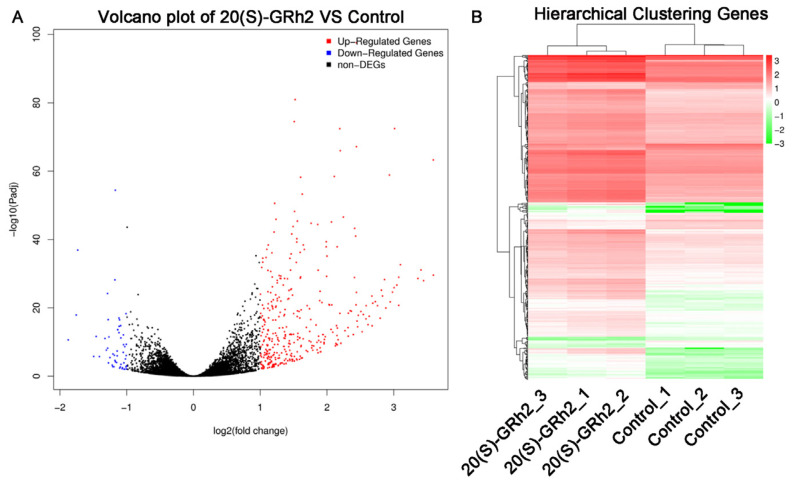
Identification of DEGs in 20(S)-GRh2-treated HeLa cells. (**A**) Volcano plots of DEGs are displayed using |log 2(Fold Change)| > 1 and adjusted *p*-value < 0.05. Red and blue dots mark upregulated genes and downregulated genes, respectively. (**B**) Cluster heatmap of DEGs with |log 2(Fold Change)| > 1 and adjusted *p*-value < 0.05. Red, upregulated DEGs; green, downregulated DEGs; DEGs, differentially expressed genes; 20(S)-GRh2, 20(S)-ginsenoside Rh2.

To clarify the potential mechanism by which 20(S)-GRh2 induces HeLa cell apoptosis, we explored DEGs by conducting RNA sequencing on HeLa cells treated with 45 μM 20(S)-GRh2 and control. We screened 361 upregulated DEGs and 53 downregulated DEGs with the criteria of |log2(Fold Change)| > 1 and *p* < 0.05 (Figure 2A). Cluster analysis of the DEGs, as shown in Figure 2B, demonstrated that biological replicates in each group have great homogeneity.

### 2.3. Enrichment Analysis of DEGs

**Figure 3:**
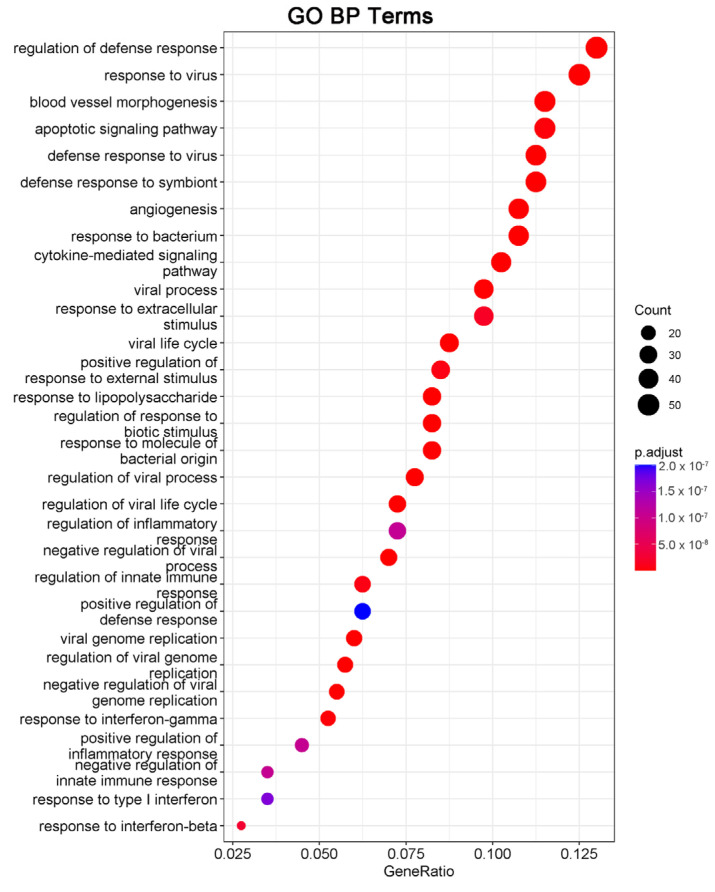
DEGs in 20(S)-GRh2-treated HeLa cells were analyzed by GO functional enrichment, and the top 30 functionally enriched terms annotated to BP are shown. GO, gene ontology; BP, biological processes; DEGs, differentially expressed genes; 20(S)-GRh2, 20(S)-ginsenoside Rh2.

**Figure 4:**
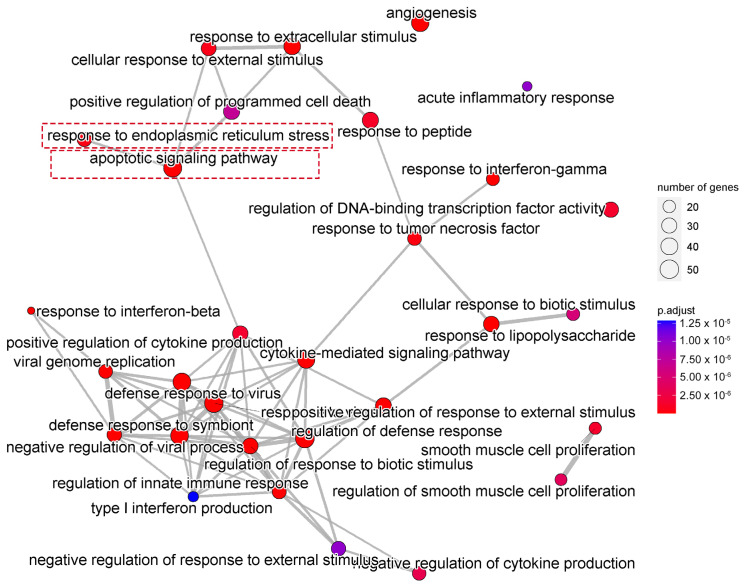
Network showing the overlapping gene sets among the GO-BP terms after removing redundant genes. GO, gene ontology; BP, biological processes.

We next performed gene ontology (GO) analysis on the DEGs of 20(S)-GRh2-treated HeLa cells to identify the terms related to biological processes (BP). The top 30 GO BP terms are shown in a bubble plot in Figure 3. The DEGs were mainly enriched in the regulation of defense response, response to virus, blood vessel morphogenesis, and apoptotic signaling pathway. Figure 4 displays the network of the similarity between BP enrichment terms and shows that the apoptotic signaling pathway was closely related to response to endoplasmic reticulum stress (ERs).

**Figure 5:**
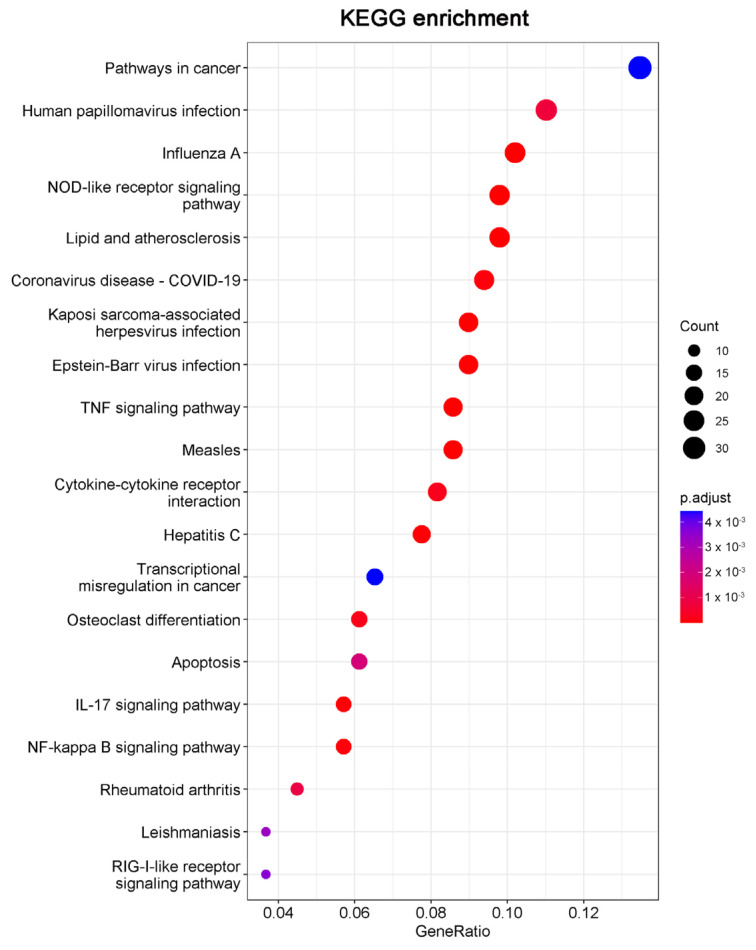
The top 20 pathways in the KEGG pathway enrichment analysis of the DEGs in 20(S)-GRh2-treated HeLa cells are shown in bubble plots. DEGs, differentially expressed genes; KEGG, Kyoto Encyclopedia of Genes and Genomes; 20(S)-GRh2, 20(S)-ginsenoside Rh2.

Consistent with the GO results, Kyoto Encyclopedia of Genes and Genomes (KEGG) enrichment analysis also revealed DEGs were relevant with apoptotic pathways (Figure 5). The 15 DEGs enriched in the apoptosis pathway are listed in Table 1, including 14 significantly upregulated genes (FOS, JUN, ATF4, DDIT3, PMAIP1, BBC3, NFKBIA, TNFSF10, FAS, ERN1, BIRC3, TRAF1, GADD45A, and CTSS genes) and one significantly downregulated gene (TUBA4A gene). These results indicated that 20(S)-GRh2 may exhibit its antitumor activity by regulating these apoptosis-related genes.

### 2.4. Protein–Protein Interaction (PPI) Network Construction and Hub Gene Screening

**Figure 6:**
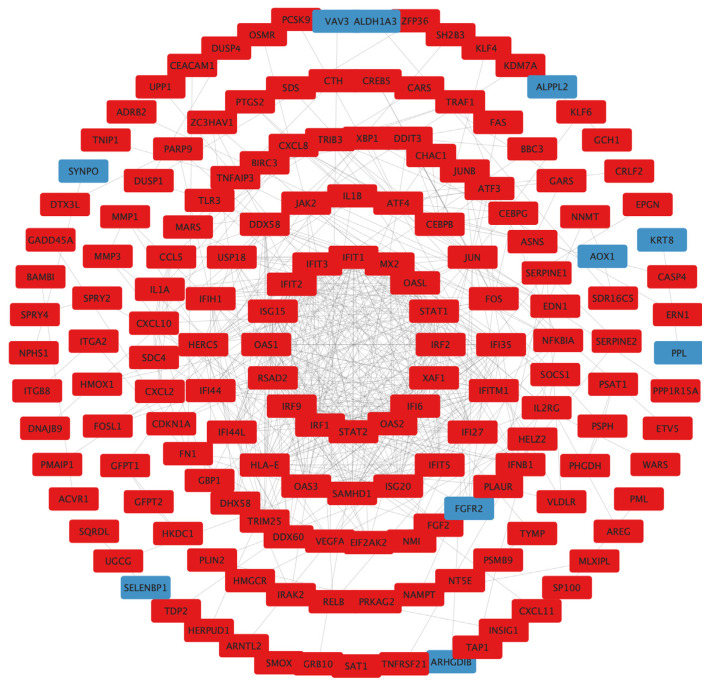
PPI network construction. Circle size reflects importance in the network (a smaller circle indicates more importance in the network). Red mark, upregulated DEGs; blue mark, downregulated DEGs; PPI, protein–protein interaction; DEGs, differentially expressed genes.

**Figure 7:**
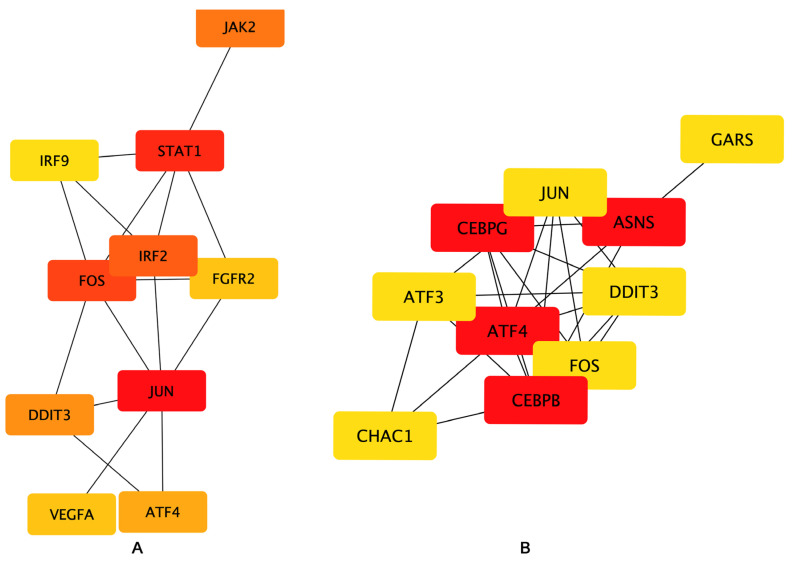
Hub genes network. The top 10 hub genes of the (**A**) Bottleneck and (**B**) EcCentricity algorithm were screened by the CytoHubba plugin. Higher rankings are indicated by deeper red colors.

To examine the interaction among the DEGs, a PPI network was constructed using the STRING database, and the results were visualized using Cytoscape software (Figure 6), followed by hub genes-screening in Figure 7. Among the top 10 hub genes, 4 genes (JUN, FOS, ATF4, and DDIT3 genes) belonged to the apoptosis pathway.

### 2.5. qPCR Verification

**Figure 8:**
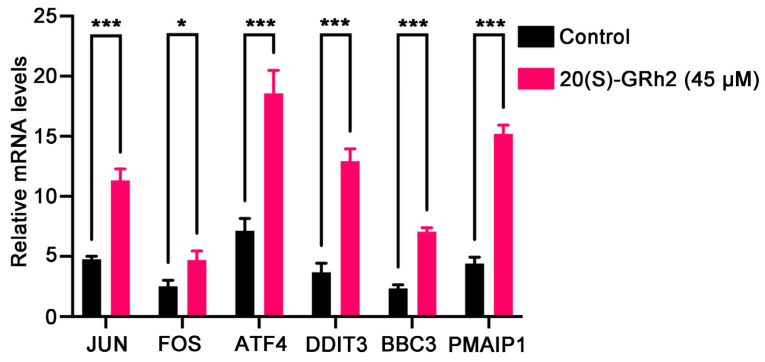
Validation of selected DEGs by qPCR. The mRNA expression levels of the DEGs were quantified in control and 45 μM 20(S)-GRh2 groups by qPCR. GAPDH mRNA was used as loading control. Data presented as mean ± standard deviation of three independent experiments (two-tailed Student’s *t*-test). * *p* < 0.05; *** *p* < 0.001 vs. control. DEGs, differentially expressed genes; 20(S)-GRh2, 20(S)-ginsenoside Rh2.

We next examined the mRNA expression of known genes deeply involved in apoptosis including JUN, FOS, ATF4, DDIT3, BBC3, and PMAIP1 in 20(S)-GRh2-treated HeLa cells by qPCR (Figure 8). The results were in line with the RNA-Seq data, in which these apoptosis-relevant genes were upregulated after 20(S)-GRh2 treatment and which indicated that the RNA sequencing data were reliable.

### 2.6. Differential Transcript Usage (DTU) Analysis

**Figure 9:**
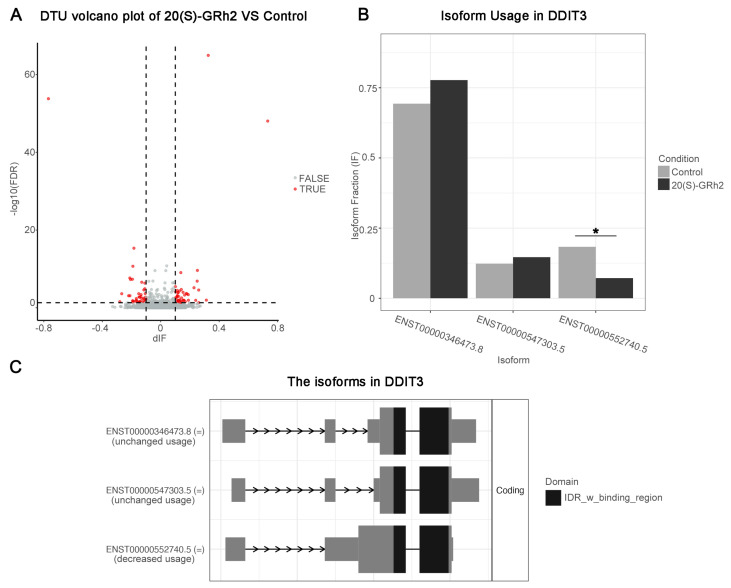
DTU analysis. (**A**) Volcano plots showing genes with DTU (red dots mark DTU; differential isoform fraction (dIF) > 0.05 and FDR <  0.05) in the control and G-Rh2-treated groups. (**B**) Quantitation of the isoform fraction in control and 20(S)-GRh2 treated groups. (**C**) Isoform landscape of all 3 isoforms of DDIT3 in 20(S)-GRh2 treatment group compared with control. * *p* < 0.05. 20(S)-GRh2, 20(S)-ginsenoside Rh2.

Significant gene expression modifications may occur at the transcriptional level and are ignored in classical DEG analysis [13]. To overcome this limitation, we used DTU analysis to characterize isomeric differences, which in turn measure the relative contribution of transcripts to overall gene expression. The transcript abundance was quantified using Kallisto, and 86 differential transcript usage events were identified by the IsoformSwitch R package (Figure 9A, Table S1). Notably, the usage of short isoform (ENST00000552740.5) in DDIT3, also as a differential transcript, was significantly reduced after 20(S)-GRh2 treatment (Figure 9B). The domain result in isoform landscape of DDIT3 showed that all three isoforms have an IDR _w_ binding region domain, and the domain of the short isoform is closer to the coding end. In addition, the short isoform has a shorter exon length near the end of the coding region (Figure 9C).

### 2.7. Molecular Docking

**Figure 10:**
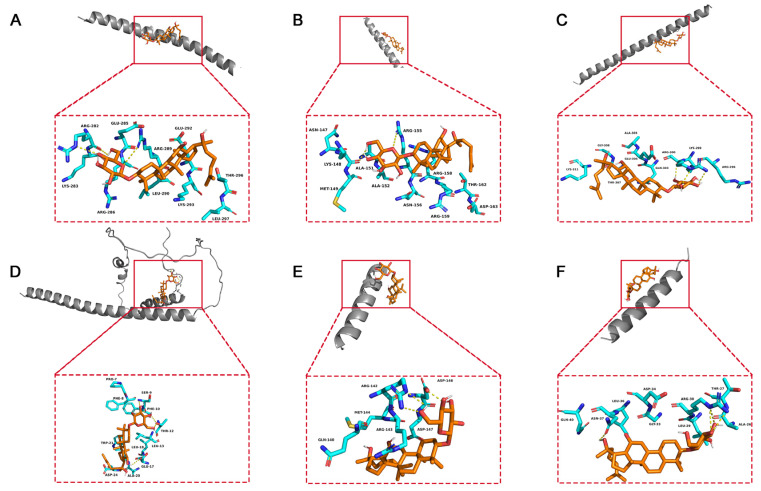
Molecular docking models of G-Rh2 and (**A**) JUN, (**B**) FOS, (**C**) ATF4, (**D**) DDIT3, (**E**) BBC3, and (**F**) PMAIP1. 20(S)-GRh2, 20(S)-ginsenoside Rh2.

To elucidate the binding mode of 20(S)-GRh2 for their targets (JUN, FOS, ATF4, DDIT3, BBC3, and PMAIP1), molecular docking analysis was performed. The results showed that 20(S)-GRh2 is bound to its residues of targets primarily through hydrogen-bonding interactions and weak interactions (Figure 10 and Table 2). Furthermore, the active sites of each target were occupied successfully by G-Rh2. The binding energy for 20(S)-GRh2-target complexes are lower than −5.0 kcal/mol, indicating highly stable binding.

## 3. Discussion

In this study, a HeLa cell line was used to evaluate the effect of 20(S)-GRh2 antihuman cervical cancer in vitro. Our results showed that 20(S)-GRh2 upregulated ATF4, DDIT3, JUN, FOS, BBC3, and PMAIP1 gene expression, as well as significantly inhibited the isoform (ENST00000552740.5) usage of the transcript DDIT3 to induce endoplasmic reticulum stress-related apoptosis in HeLa cells. Our research provided novel evidence for the mechanism of 20(S)-GRh2 anticervical cancer.

In accordance with previous results [14], we found that 20(S)-GRh2 inhibited the cell viability of HeLa in a dose–time parallel dependent manner. Because rapid proliferation and apoptosis escape are important hallmarks of tumor cells [15], we next mainly focused on the roles and targets of 20(S)-GRh2 in apoptotic events. We found that 20(S)-GRh2 induced apoptosis in HeLa cells with increased caspase 3 activity and phosphatidylserine eversion, which is consistent with its induction of apoptotic events in multiple cancers [16,17].

To understand the molecular mechanism of 20(S)-GRh2 in cervical cancer, we collected HeLa cells administrated with or without 20(S)-GRh2 for RNA-seq and found that upregulated genes after 20(S)-GRh2 treatment were functionally annotated with apoptosis pathways. Additionally, the apoptosis might be mainly related with response to endoplasmic reticulum stress (ERs). In addition, combined with PPI and hub gene network analysis, we found that the upregulation of ATF4, DDIT3, JUN, FOS, BBC3, and PMAIP1 gene expression is closely related to apoptosis. Given that sustained activation of ERs can trigger downstream apoptotic signaling pathways, targeting ERs-related genes has emerged as a potential strategy for cancer therapy [18,19]. Indeed, ATF4 acts as a pivotal mediator of ERs-induced apoptosis, and its translocation into the nucleus promotes ER protein folding and adaptation to stress by inducing the expression of UPR target genes such as the DDIT3 gene [20,21,22]. A similar result was also reported in lung cancer that showed that 20(S)-GRh2 triggers ER stress-related ATF4/DDIT3-induced apoptosis [23]. Although, DDIT3 is not expressed or weakly expressed under normal physiology but rapidly increases after severe or long-term ERs induction [24]. Induction of DDIT3 is strongly associated with the initiation of apoptosis associated with ERs, and silencing DDIT3 expression protects cells from apoptosis in chronic ERs [25]. In addition, DDIT3 transcriptionally regulates major apoptosis mediators of the Bcl-2 protein family, such as BBC3 and PMAIP1 [26,27]. BBC3 and PMAIP1 attach to the mitochondrial membrane through inhibiting the antiapoptotic Bcl-2 family members and activate proapoptotic Bax/Bak to enhance the permeability of the mitochondrial membrane, thereby inducing the apoptotic mitochondrial pathway [28,29]. A previous study stated that ATF4 is an important regulator of PMAIP1 transcription and promotes apoptosis in HeLa cells [30]. A recent report revealed that DDIT3 and the JUN/FOS heterodimer (AP-1 complex) jointly mediate BBC3-induced apoptosis, especially the physical association with JUN [31,32,33]. It implies that ERs-related genes (ATF4 and DDIT3) and downstream apoptosis genes (JUN, FOS, BBC3, and PMAIP1) are the potential targets of 20(S)-GRh2 and might be interacting with each other to be involved in the apoptosis induction in cervical cancer cells.

In addition, DTU analysis was performed to comprehensively analyze RNA-seq datasets at the level of transcripts. Importantly, DDIT3 is also as a differential transcript after 20(S)-GRh2 treatment, and its three isoforms all have the same IDR (intrinsically disordered regions)_w _binding region domain at the *N*-terminus. Recent studies have shown that IDR induces protein liquid–liquid phase transition and is involved in oncogene activation and carcinogenesis [34,35]. Furthermore, the IDR_w_binding region domain of the differential transcript isoform ENST00000552740.5 in DDIT3 exposed to 20(S)-GRh2 is closer to the end of the coding region; however, whether this phenotype is directly linked to the cervical cancer apoptosis induced by 20(S)-GRh2 remains to be investigated. A recent study showed that the development of cancer is accompanied by aberrant changes in many splicing events, and its exons are biased toward shorter lengths [36]. Notably, we found that ENST00000552740.5 had a shorter exon length near the end of the coding region and its usage was significantly reduced after 20(S)-GRh2 exposure. These results suggested that modulation of the DDIT3 isoform switch by 20(S)-GRh2 treatment may be a potential therapeutic strategy for anticervical cancer.

Considering the established association between apoptosis-related targets and 20(S)-GRh2, we also investigated the binding activity between the two using molecular docking. It has been reported that hydrogen bonds and weak interactions are the major contributions to strengthening the drug–target docking state [37,38]. In this study, G-Rh2 exhibited high-affinity activity with targets (ATF4, DDIT3, JUN, FOS, BBC3, and PMAIP1), binding to the residues of the targets through hydrogen bonds and weak interactions. These results further supported that apoptosis in 20(S)-GRh2-exposed HeLa cells is induced by ER stress-related genes ATF4 and DDIT3 and downstream apoptosis genes JUN, FOS, BBC3, and PMAIP1.

**Figure 11:**
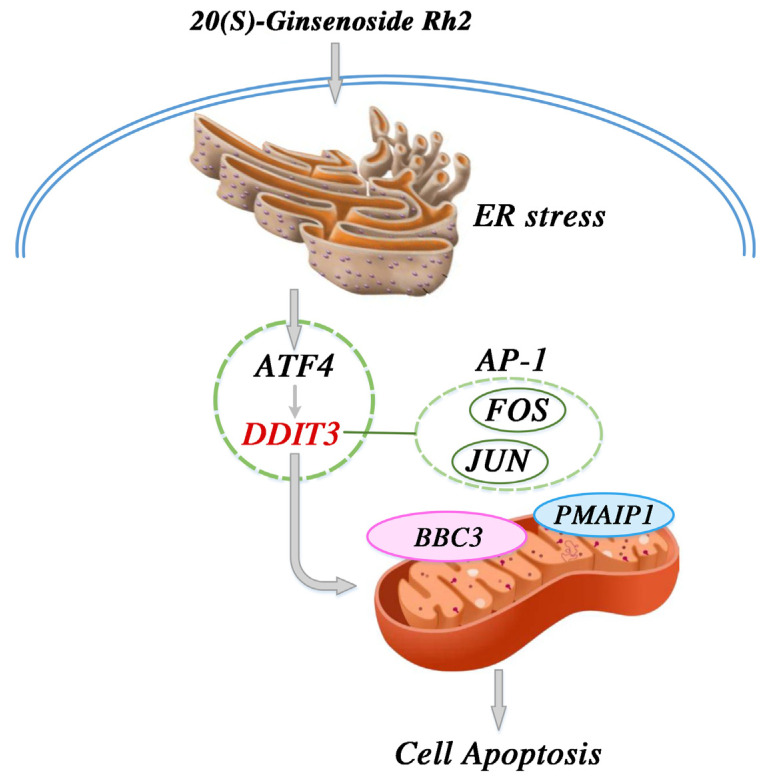
Proposed signal pathway for the anticervical cancer mechanism of 20(S)-GRh2. 20(S)-GRh2, 20(S)-ginsenoside Rh2.

From our results and the above findings, we propose a potential signaling pathway model to help in understanding the mechanism of 20(S)-GRh2-induced apoptosis in HeLa cells. We suggest that increased DDIT3 is the consequence of the induction of ERs and occurs following activation of ATF4 after 20(S)-GRh2 treatment. The activation of DDIT3 is also accompanied with the activation of BBC3 and PMAIP1. Moreover, we proposed that DDIT3 collaborates with JUN and FOS in the AP-1 complex to mediate BBC3-induced apoptosis. In summary, JUN, FOS, BBC3, and PMAIP1 may be key downstream targets of 20(S)-GRh2-induced HeLa apoptosis, as all are regulated by an ERs-mediated cascade of ATF4 and DDIT3 (summarized in Figure 11). Notably, the proposed signaling pathway model is still a hypothesis, and more research is required to determine the specific mechanism of the 20(S)-GRh2-related apoptosis pathway.

## 4. Materials and Methods

### 4.1. Cell Lines

HeLa cells (CC cell line) were purchased from the Cell Bank of the Chinese Academy of Sciences (Shanghai, China). Cells were cultured in high glucose DMEM (Hyclone, South Logan, UT, USA) with 10% fetal bovine serum (FBS) (Hyclone) and 1% penicillin-streptomycin (100 U/mL) (Hyclone) in a humidified incubator (Thermo Scientific, Waltham, MA, USA) at 37 °C in 5% CO_2._

### 4.2. Cell Viability Assay

HeLa cells were plated at 2 × 10^4^ cells/well in a six-well plate and treated with 20(S)-GRh2 (25, 45, and 65 μM, Must Bio-Technology Co., Ltd., Chengdu, China) for 12, 24, and 36 h. Cell viability was conducted using CCK-8 kits (Boster, Wuhan, China) on an Infinite M200 PRO plate reader (Tecan, Männedorf, Switzerland) at 450 nm. The 50% inhibitory concentration (IC_50_) at 24 h was calculated according to the values of cell viability [39].

### 4.3. Cell Apoptosis Analysis

The Live Cell Caspase-3 Activity and Annexin V Apoptosis Detection Kit (Beyotime, Shanghai, China) were used to evaluate HeLa cell apoptosis. Briefly, HeLa cells were cultured in a 12-well plate for 24 h at 37 °C and then treated with 20(S)-GRh2 (45 μM) for 24 h. HeLa cells were then incubated with Caspase-3 substrate (5 μM) and Annexin V–mCherry (5 μM) for 30 min in the dark at room temperature. The cells were washed twice with PBS and analyzed immediately under an EVOS FL autofluorescence microscope (Life Technologies, Carlsbad, CA, USA).

### 4.4. RNA-Seq Analysis

HeLa cells were treated with 20(S)-GRh2 (45 μM) or 0.1% *v*/*v* DMSO for 24 h. Total RNA was collected with an RNA simple extraction kit (Tiangen, Beijing, China). RNA samples were sent to the BGI Company (https://www.bgi.com/ (accessed on 30 July 2020)) (Shenzhen, China), and samples (5 mg) were used for library construction using the TruSeq RNA Sample Preparation Kit (Illumina, San Diego, CA, USA). Libraries were quantified by the Agilent 2100 Bioanalyzer (Agilent Technologies, Palo Alto, CA, USA) and sequenced on an Illumina HiSeq 4000 platform (Illumina, San Diego, CA, USA).

### 4.5. DEG Screening and Functional Enrichment Analysis

The DEGs between control and 45 μM 20(S)-GRh2 groups were screened using DESeq2. We set the screening condition as |log 2(Fold Change)| > 1 and *p* adjusted value < 0.05. Gene ontology (GO) categories and Kyoto Encyclopedia of Genes and Genomes (KEGG) pathways enrichment analysis of DEGs were conducted using DAVID database and visualized by the “Cluster Profiler” of R.

### 4.6. Protein–Protein Interaction (PPI) Network and Hub Gene Analysis

The PPI network of DEGs (connection score > 0.9) was built using the STRING database (https://string-db.org/ (accessed on 7 February 2022)) and visualized using Cytoscape. The top 10 hub genes were screened by the CytoHubba plug-in with the Bottleneck and EcCentricity algorithm [40,41].

### 4.7. qPCR Analysis

Total RNA was extracted using an RNA simple extraction kit (Tiangen) and then reverse transcribed into cDNA using the Primescript RT kit (Takara) following the manufacturer’s instructions. The TB Green Premix Ex Taq kit (Takara) was used for qPCR quantification. The relative expression of genes was quantified by the 2^−ΔΔCT^ method [42]. GAPDH mRNA was an internal control. Primer sequences were as follows: PMAIP1_forward: 5′-GTGCCCTTGGAAACGGAAGA-3′, reverse: 5′-CCAGCCGCCCAGTCTAATCA-3′; ATF4_forward: 5′-CACTAGGTACCGCCAGAAGA-3′, reverse: 5′-AATCCGCCCTCTCTTTTAGA-3′; BBC3_forward: 5′-CTGTGAATCCTGTGCTCTGC-3′, reverse: 5′-AATGAATGCCAGTGGTCACA-3′; DDIT3_forward: 5′-GGAAACAGAGTGGTCATTCCC-3′, reverse: 5′-CTGCTTGAGCCGTT CATTCTC-3′; JUN_forward: 5′-TCCAAGTGCCGAAAAAGGAAG-3′; reverse: 5′-CGAGTTCTGAGCTTTCAAGGT-3′; FOS_forward: 5′-CCGGGGATAGCCTCTCTTACT-3′; reverse: 5′-CCAGGTCCGTGCAGAAGTC-3′ and GAPDH_forward: 5′-ACAACTTTGGTATCGTGGAAGG-3′, reverse: 5′-GCCATCACGCCACAGTTTC-3′.

### 4.8. Differential Transcript Usage (DTU) Analysis

Raw fastq files from RNA-seq datasets were processed with Trimmomatic to remove adapters and low-quality reads, followed by quantification at the transcript level by Kallisto with default settings [43]. For differential transcript usage (DTU) analysis, the Kallisto transcript abundance table was applied as input to import Rdata function of R package IsoformSwitchAnalyzeR to create a switchAnalyzeRlist object. Then, we set a cutoff (gene expression > 10 and isoform expression > 3 and differential isoform fraction (dIF) > 0.05) to filter out unqualified genes and isoforms. The single isoform genes were also removed. To test differential transcript usage, the IsoformSwitchTestDEXseq function was employed, and those isoforms with dIF > 0.05 and FDR < 0.05 were considered to be significant. The transcript isoform landscape was visualized with the switchplot function of IsoformSwitchAnalyzeR.

### 4.9. Molecular Docking

The 3D structure of proteins was downloaded from Protein Data Bank (PDB, https://www.rcsb.org/ (accessed on 28 September 2022)) and AlphaFold protein structure databases (https://alphafold.ebi.ac.uk/ (accessed on 28 September 2022)), including JUN (PDB ID: 1S9K Entity 5), FOS (PDB ID: 1S9K Entity 4), ATF4 (PDB ID: 1CI6 Entity 1), DDIT3 (Alphafold ID: AF-P35638-F1), BBC3 (PDB ID: 6QG8 Entity 2), and PMAIP1(PDB ID: 3MQP Entity 2). The 3D structure of 20(S)-GRh2 was downloaded from PubChem (PubChem CID 119307). Finally, molecular docking for G-Rh2 and proteins were performed with AutoDock 4.0 and visualized with Pymol.

### 4.10. Statistical Analysis

Statistical analysis was performed using GraphPad Prism 9.0 with experiments that were independently repeated three times. Ordinary two-way ANOVA followed by Tukey’s post-hoc test or two-tailed Student’s *t*-test were applied to compare 20(S)-GRh2 and control groups. *p* <0.05 was considered significant.

## 5. Conclusions

In the present study, we found that 20(S)-GRh2 inhibited cell proliferation and induced cell apoptosis in HeLa cells via upregulating with ERs-related genes (ATF4, DDIT3) and its downstream apoptosis genes (JUN, FOS, BBC3, and PMAIP1). These findings indicated that 20(S)-GRh2 may be a potential drug for CC treatment. A limitation of our study was that the prediction mechanism was not validated in vivo. Further studies are needed to better understand the anticancer mechanism of 20(S)-GRh2 in vivo. Despite the limitation, our study identified potential target genes induced by 20(S)-GRh2 related to the apoptosis of HeLa that may expand sights for the treatment of CC.

## Figures and Tables

**Table 1:** DEGs in apoptosis pathways.

Gene ID	Symbol	Log2(FC)	*p*-Value	Regulated by 20(S)-GRh2
3725	JUN	1.99	2.18 × 10^−32^	UP
2353	FOS	1.12	8.21 × 10^−8^	UP
468	ATF4	1.21	1.11 × 10^−45^	UP
1649	DDIT3	2.14	2.22 × 10^−17^	UP
27113	BBC3	1.69	7.08 × 10^−32^	UP
5366	PMAIP1	1.97	5.99 × 10^−14^	UP
2081	ERN1	1.02	4.77 × 10^−6^	UP
8743	TNFSF10	1.27	5.53 × 10^−5^	UP
355	FAS	1.07	5.23 × 10^−9^	UP
4792	NFKBIA	1.04	3.89 × 10^−17^	UP
1647	GADD45A	1.48	1.26 × 10^−26^	UP
330	BIRC3	1.39	5.20 × 10^−17^	UP
1520	CTSS	1.23	1.96 × 10^−5^	UP
7185	TRAF1	3.05	1.12 × 10^−29^	UP
7277	TUBA4A	−1.12	1.10 × 10^−19^	Down

**Table 2:** Molecular docking results.

Target Name	Docking Score (Kcal/M)	Residues Involved in Hydrogen	Residues Involved in Weak Interactions
JUN	−5.8	ARG282; ARG289	LYS283; GLU285; ARG286; LEU290; GLU292; LYS293; THE296; LEU297
DDIT3	−5.6	SER9; PHE10; THR12; GLU17	PRO7; PHE8; LEU13; LEU18; ALA20; TRP21; ASP24
ATF4	−5.6	LYS299; ARG300	ASN147; MET149; ALA151; ALA152; ASN156; ARG158; ARG159; THR162; ASP163
FOS	−5.6	LYS148; ARG155	ARG296; GLN303; GLU304; ALA305; THR307; GLY308; LYS311
PMAIP1	−5.2	ARG30	ALA26; THR27; LEU29; GLY33; ASP34; LEU36; ASN37; GLN40
BBC3	−5.0	ARG142; ARG146	GLN140; ARG143; MET144; ASP147

## Data Availability

The datasets generated for this study can be found in the NCBI SRA accession (BioProject ID: PRJNA846264, https://www.ncbi.nlm.nih.gov/sra/PRJNA846264 (accessed on 6 June 2022)).

## Supplementary Materials

The following supporting information can be downloaded at: https://www.mdpi.com/article/10.3390/molecules27227865/s1, Table S1: DTU_result.

## Sample Availability

Samples of some reported nanomaterials are available from the authors upon request.

## Abbreviations

Ginsenoside Rh2	GRh2
DEGs	Differentially expressed genes
ERs	Endoplasmic reticulum stress
GO	Gene ontology
KEGG	Kyoto Encyclopedia of Genes and Genomes
CC	Cervical cancer
FBS	Fetal bovine serum
PS	Phosphatidylserine
BP	Biological process
